# The Bacillus

**Published:** 1883-01

**Authors:** 


### The Bacillus.

Some one has said that "history repeats itself." This aphorism, in some respects, is just as applicable in the history of science as in the history of society.

Medicine has its "new departures" and fashions as surely as any other factor in human events. This, of course, is more commonly so in "æsthetic medicine—homœpathy—but occasionally it reaches the scientist.

It was the fashion in the primitive days of medicine to represent disease as an indwelling foe, a mighty homunculus, who seized upon the vital forces within the body, controlled their action, traversed the great avenues of circulation and besieged the very citadel of the soul itself. and if not defeated in time, the patient must die. Succeeding this came the age of humors. Then special influences, where each organ was governed by its particular Deity. Then inflammation was the sole cause of all human infirmities. And now we return again to the original idea, that of the personification of disease. Hence it is the fashion to hunt out this micr scopic enemy, define his shape, size and habits of life. Give him a name, and then, if his style is not after our ideas of propriety, educate and civilize him, that he may become a potent soldier to war against his barbarous ancestors.

Jenner "builded wiser than he knew" when he tamed the viscious spirit of small-pox virus and made it a protecting agent against that dreaded disease.

Commencing with his experiments, the idea of the bacillus has gradually engrafted itself upon the minds of medical investigators, until to-day we have it claimed that the great catalogue of the most serious diseases known to men are produced by the presence of bacilli in the body.

Salisbury years ago gave us the bacillus of malaria, and Crudelli, from Italy, adds his testimony to confirm the theory. From Germany we have bacillus typhosus—Clebs; from France bacillus anthracis—Pasteur; from Berlin bacillus tuberculosis—Koch; from Philadelphia bacillus of diptheria—Wood and Formad; and from Chicago bacillus of swine-plague—Detmers, with several others to hear from.

The experimental researches by these experienced and careful investigators have been so conducted as to inspire the confidence of the profession, and have no doubt established the fact that the presence of certain parasites is really the cause of certain forms of disease. Yet it is well for us to remember that there are other forces acting to undermine the health of our people. Sir Charles Lamb once remarked, that "we often laugh at the folly exhibited in a large flock of sheep, by their great haste to jump a fence, just because the principal sheep in the flock led the way, but we forget that we sometimes are governed by the same influences."

So in medical science. We are too apt to rush off after some new theory, just because one of the leading workers has declared his faith in that direction.

The great Vienna "Simon" (Billroth) says "thumbs up," and up goes the great surgical thumbs all over the world; and alas! for the poor stomach that must be resected. And Lister declares for spray and gauze, and he who fails to use them is not in fashion. Now, we do not wish to be understood as underestimating any of these advances, but let us remember that the saving quality of the true physician is caution.

"Prove all things and hold fast to that which is good." So with the bacillus. We should be sure of our enemy before we forsake the precepts of our fathers and their weapons of fighting disease, and when we have carefully determined between those diseases which are parasitic and those which are not, then the practical question to be investigated is, what shall we do with this microscopic enemy? But don't forget that he may be present and still not be the cause of the trouble, or you may direct your batteries against an innocent party.

Lister fences him out with spray, and oiled silk, and gauze. Declat fights him with phenic acid, while Pasteur captures the little demon, civilizes and domesticates him, and makes him a useful member of society.

Each method, no donbt, has its proper place, and we have every reason to believe that progressive investigators will separate the truth from error in all this work, and when the pathology is clearly established the mode of action will soon be well defined.

---

Anæsthesia of the Pharynx.—According to M. Cozal, painting the mucous membrane of the pharynx with the tincture of coca will secure anæsthesia of the part. If this be true, the result is probably due to the alcohol.

